# Analysis of the transcriptional activity of model piggyBac transgenes stably integrated into different loci of the genome of CHO cells in the absence of selection pressure

**DOI:** 10.18699/VJGB-23-105

**Published:** 2023-12

**Authors:** L.A. Yarinich, A.A. Ogienko, A.V. Pindyurin, E.S. Omelina

**Affiliations:** Institute of Molecular and Cellular Biology of the Siberian Branch of the Russian Academy of Sciences, Novosibirsk, Russia; Institute of Molecular and Cellular Biology of the Siberian Branch of the Russian Academy of Sciences, Novosibirsk, Russia; Institute of Molecular and Cellular Biology of the Siberian Branch of the Russian Academy of Sciences, Novosibirsk, Russia; Institute of Molecular and Cellular Biology of the Siberian Branch of the Russian Academy of Sciences, Novosibirsk, Russia

**Keywords:** TRIP, barcode, chromatin position effect, transgene, chromatin, transcription, TRIP, штрихкод, эффект положения гена, трансген, хроматин, транскрипция

## Abstract

CHO cells are most commonly used for the synthesis of recombinant proteins in biopharmaceutical production. When stable producer cell lines are obtained, the locus of transgene integration into the genome has a great influence on the level of its expression. Therefore, the identification of genomic loci ensuring a high level of protein production is very important. Here, we used the TRIP assay to study the influence of the local chromatin environment on the activity of transgenes in CHO cells. For this purpose, reporter constructs encoding eGFP under the control of four promoters were stably integrated into the genome of CHO cells using the piggyBac transposon. Each individual transgene contained a unique tag, a DNA barcode, and the resulting polyclonal cell population was cultured for almost a month without any selection. Next, using the high-throughput sequencing, genomic localizations of barcodes, as well as their abundances in the population and transcriptional activities were identified. In total, ~640 transgenes more or less evenly distributed across all chromosomes of CHO cells were characterized. More than half of the transgenes were completely silent. The most active transgenes were identified to be inserted in gene promoters and 5’ UTRs. Transgenes carrying Chinese hamster full-length promoter of the EF-1α gene showed the highest activity. Transgenes with a truncated version of the same promoter and with the mouse PGK gene promoter were on average 10 and 19 times less active, respectively. In total, combinations of genomic loci of CHO cells and transgene promoters that together provide different levels of transcriptional activity of the model reporter construct were described.

## Abstract (ru)

Культивируемые клетки яичника китайского хомячка (CHO) наиболее часто используются для синтеза рекомбинантных белков в биофармацевтическом производстве. При получении стабильных клеточных линий-продуцентов локус интеграции трансгена в геном оказывает большое влияние на уровень его экспрессии (явление, известное как эффект положения гена). Соответственно, поиск локусов генома, обеспечивающих высокий уровень продукции белков, является актуальной практической задачей. В данной работе мы использовали метод TRIP для исследования влияния локального окружения хроматина на активность трансгенов, встроенных в разные локусы генома культивируемых клеток CHO. С этой целью репортерные конструкции, кодирующие белок eGFP под контролем четырех разных промоторов, были стабильно встроены в геном клеток CHO при помощи транспозона piggyBac. При этом каждый отдельный трансген содержал уникальную метку – ДНК-штрихкод. Полученная трансгенная поликлональная популяция клеток была культивирована в течение месяца без какой-либо селекции. Далее при помощи присутствующих в конструкциях штрихкодов и высокопроизводительного секвенирования были определены сайты локализации трансгенов в геноме, измерена их представленность в популяции, а также транскрипционная активность. Всего удалось полностью охарактеризовать около 640 трансгенов, более-менее равномерно распределенных по всем хромосомам клеток CHO. Более половины трансгенов оказались полностью молчащими. Наиболее активные трансгены выявлены в окрестностях геномных сайтов инициации транскрипции – в промоторных и 5’-некодирующих районах генов. Наибольшей активностью обладали трансгены, несущие полноразмерный промотор гена EF-1α китайского хомячка. Трансгены с укороченным вариантом этого же промотора, а также трансгены с промотором мышиного гена PGK (mPGK) были соответственно в среднем в 10 и 19 раз менее активны. В целом в результате данной работы выявлены сочетания локусов генома культивируемых клеток CHO и промоторных элементов, которые обеспечивают разные уровни транскрипционной активности модельной репортерной конструкции.

## Introduction

The TRIP (thousands of reporters integrated in parallel) assay enables large-scale studies of the influence of the chromatin position effects on the transgene activity. It is based on DNA barcodes (hereafter, barcodes) and was originally performed on the mouse embryonic stem cells using the piggyBac transposon system to deliver reporter transgenes into the genome (Akhtar et al., 2013). A barcode is a short DNA sequence (16–20 bp) that is unique to each individual transgene in the study. It should be noticed that the barcode is located within the transcribed region of the transgenes ensuring its presence not only in DNA, but also in mRNA molecules. Therefore, the barcode can be used for quantitative measurements of the level of transcriptional activity of transgenes

The piggyBac transposon system has been previously used to effectively modify various cell lines and organisms (Wilson et al., 2007) even with large transgenic constructs (Ding et al., 2005). In addition, the piggyBac transposon is characterized by a relatively uniform distribution of insertions across chromosomes (Huang et al., 2010). In TRIP experiments, the system for transgenesis consists of two plasmid constructs: a construct for the expression of piggyBac transposase, which catalyzes the insertion of a transgene into a random genome locus, and the transgene itself – a target construct (consisting of a promoter, a reporter gene, a barcode, and a polyadenylation signal) located between the inverted terminal repeats of the piggyBac transposon (Akhtar et al., 2014; Lebedev et al., 2019). Co-transfection of cells with such plasmid constructs enables obtaining a polyclonal population of transgenic cells, in which each individual transgene insertion in the genome is marked with a unique barcode sequence. After cultivation of transfected cells, genomic DNA and total RNA are isolated from them. Using the genomic DNA sample, the genomic localization of each transgene is identified and the abundance of each barcode in the cell population is measured. Based on the total RNA sample, the abundance of each barcode in the total pool of transcripts synthesized from transgenes is measured. Finally, the ratio of the abundance of each barcode in mRNA molecules to its abundance in the cell population allows quantitative estimation of transcriptional activity of all transgenes (Akhtar et al., 2014).

In this study, we used the TRIP assay to investigate the chromatin position effects on the transcriptional activity of stably integrated transgenes in the Chinese hamster ovary cells (CHO). The CHO line is the most commonly used cell line to produce a variety of proteins (Xu et al., 2023). Despite the availability of other mammalian cells, such as baby hamster kidney cells, murine myeloma NS0 cells, human embryonic kidney cells (HEK293), human embryonic retinal PerC6 cells, more than 70 % of all recombinant therapeutic proteins are produced in CHO cells (Kim et al., 2012; Ritacco et al., 2018; Gupta et al., 2021). The popularity of CHO cells is explained by the following reasons. First, the use of CHO cells for the production of recombinant proteins is safe, since CHO cells are insensitive to infection by human viruses (Lalonde, Durocher, 2017). Second, CHO cells have the ability for efficient posttranslational modification and produce recombinant proteins in human-compatible glycoforms (Stache et al., 2019). Third, CHO cells have a high growth rate and are relatively easily adapted to growth in suspension, which is a preferred characteristic for large-scale cultivation in bioreactors (Ritacco et al., 2018; Dahodwala, Lee, 2019). Currently, bioreactors with a volume of more than 10 thousand liters are used for suspension cultures of recombinant CHO cells producing therapeutic antibodies (Kim et al., 2012).

Localization in the genome has a great influence on the expression level of the recombinant gene (a phenomenon known as the chromatin position effect) (Gierman et al., 2007; Babenko et al., 2010; Ruf et al., 2011; Chen M. et al., 2013; Elgin, Reuter, 2013). Integration into inactive heterochromatin results in low or no transgene expression, whereas integration into active euchromatin often allows moderate to high transgene expression. However, simple integration into euchromatin may not be sufficient to ensure long-term expression of the recombinant gene. The phenomenon of transgene expression silencing is well known in mammalian cells, it occurs in part likely due to the influence of adjacent condensed chromatin.

Thus, integration of transgenes into transcriptionally highly active regions of the genome is a reasonable strategy to avoid position effects. This study was aimed at analyzing the transcriptional activity of the piggyBac transgenes integrated into different loci of the genome of CHO cells in the absence of selection pressure.

## Materials and methods

Generation of the pPB-mPGK-Puro-IRES-eGFP-PI.11- TR.242 construct. Plasmid pPB-mPGK-Puro-IRES-eGFPPI. 11-TR.242 was made based on a previously described “universal” construct (Lebedev et al., 2019). The insertion was amplified using the primers mPGK-EcoRI-F and eGFPXbaI- R (Table 1) and the plasmid pPTK-Gal4-mPGK-Puro- IRES-eGFP-sNRP-pA as a template (Akhtar et al., 2013). Fifty μl of the reaction mixture contained 1 ng of plasmid template, 10 μl of 5× Phusion HF buffer (Thermo Fisher Scientific), 1 μl of each 10 μM primer, 0.2 mM dNTPs, and 2.5 U of Phusion polymerase (Thermo Fisher Scientific). The PCR thermal cycle conditions were as follows: 98 °C for 30 sec, 35 cycles of 98 °C for 10 sec, 62 °C for 10 sec, 72 °C for 1 min, and a final incubation for 10 min at 72 °C.

**Table 1.**
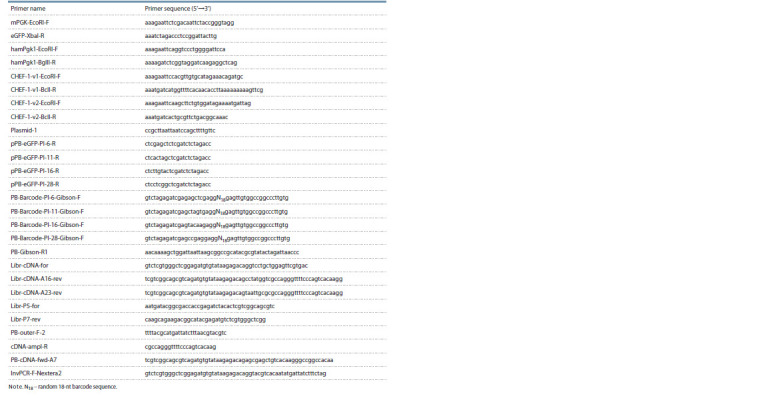
Primers used in the study Notе. N18 – random 18-nt barcode sequence.

Cloning of constructs with various Chinese hamster gene promoters. The plasmid pPB-mPGK-Puro-IRESeGFP- PI.11-TR.242 was digested with EcoRI, BglII, and AgeI restriction enzymes. To obtain inserts, the sequences of the Chinese hamster PGK gene promoter and the long and short variants of the EF-1α gene promoter were amplified using the primers hamPgk1-EcoRI-F and hamPgk1- BglII-R, CHEF-1-v1-EcoRI-F and CHEF-1-v1-BclI-R, and CHEF-1-v2-EcoRI-F and CHEF-1-v2-BclI-R (see Table 1), respectively. Fifty μl of the reaction mixture contained 50 ng of genomic DNA template isolated from CHO cells, 10 μl of 5× Phusion HF buffer (Thermo Fisher Scientific), 1 μl of each 10 μM primer, 0.2 mM dNTPs, and 2.5 U of Phusion polymerase (Thermo Fisher Scientific). The PCR thermal cycle conditions were as follows: 98 °C for 30 sec, 35 cycles of 98 °C for 10 sec, 62 °C for 10 sec, 72 °C for 1 min, and a final incubation for 10 min at 72 °C.

Generation of barcoded plasmid libraries. Barcoded plasmid libraries were made according to a previously described protocol (Lebedev et al., 2019) using the Gibson cloning method. For this purpose, vectors and inserts containing an 18-nt DNA barcode and a promoter index were prepared using PCR. For vector amplification, the primers Plasmid-1 and pPB-eGFP-PI-6-R / pPB-eGFP-PI-11-R / pPB-eGFP-PI-16-R / pPB-eGFP-PI-28-R were used (see Table 1) for the constructs with the Chinese hamster PGK gene promoter / mPGK gene promoter / short variant of the EF-1α gene promoter / long variant of the EF-1α gene promoter, respectively. The primers PB-Barcode-PI-6-Gibson-F / PB-Barcode-PI-11-Gibson-F / PB-Barcode-PI-16-Gibson-F / PB-Barcode-PI-28-Gibson-F and PB-Gibson-R1 (see Table 1) were used to amplify the barcoded inserts for constructs with the Chinese hamster PGK gene promoter / the mPGK gene promoter / short variant of the EF-1α gene promoter / long variant of the EF-1α gene promoter, respectively. Fifty μl of the reaction mixture con-tained 1 ng of template, 10 μl of 5× Phusion HF buffer (Thermo Fisher Scientific), 1 μl of each 10 μM primer, 0.2 mM dNTPs, and 2.5 U of Phusion polymerase (Thermo Fisher Scientific). The PCR thermal cycle conditions were as follows: 98 °C for 30 sec, 35 cycles of 98 °C for 10 sec, 62 °C for 10 sec, 72 °C for 1 min, and a final incubation for 10 min at 72 °C. After purification, 200 ng of “vector” and 135 ng of “inserts” were mixed with 10 μl of 2× NEBuilder HiFi DNA Assembly Master in a total volume of 20 μl. DNA ligation and bacterial transformation were performed as described previously (Lebedev et al., 2019). Barcoded plasmid libraries were isolated using the Mega Plasmid Kit (Qiagen).

Generation of polyclonal transgenic population of CHO cells. Twenty-four h before transfection, CHO-S cells (hereafter CHO cells; kindly provided by Dr. A.V. Taranin, Institute of Molecular and Cellular Biology, Novosibirsk, Russia) were seeded into a 12-well culture plate at a concentration of 1.5 × 105 cells per ml in IMDM medium supplemented with 10 % bovine serum. Cells were co-transfected with a mixture of barcoded plasmid libraries (3 μg) and the pRP[Exp]-mCherry-CAG>hyPBase plasmid (VectorBuilder #VB160216-10057; kindly provided by Prof. V.V. Verkhusha, Albert Einstein College of Medicine, Bronx, NY, USA) (0.3 μg) using the X-tremeGENE HP DNA transfection reagent (Roche). The transfected cells were cultured for a month in the absence of selection pressure.

Isolation of genomic DNA. Genomic DNA was isolated from 5 · 107 cells of the resulting polyclonal transgenic population using the PureLink® Genomic DNA Kit (Invitrogen) according to the manufacturer’s recommendations.

Isolation of total RNA, reverse transcription. Total RNA was isolated from 5 · 107 cells of the resulting polyclonal transgenic population using RNAzol RT (Molecular Research Center) according to the manufacturer’s recommendations. The isolated RNA was incubated with 20 U of DpnI restriction endonuclease (New England Biolabs) and 3 U of DNase I (Thermo Fisher Scientific) for 30 min at 37 °C. The Clean RNA Standard kit (Evrogen) was used to purify RNA. Two μg of purified total RNA was mixed with 1 μl of the 50 mM oligo(dT) primer in a total volume of 13.5 μl, and the mixture was incubated for 5 min at 65 °C. The subsequent reverse transcription reaction was carried out in a volume of 20 μl with the following components: 13.5 μl of RNA with annealed primer, 4 μl of 5× RT buffer (Thermo Fisher Scientific), 1 μl of 10 mM dNTPs, 1 μl of RNaseOUT (Thermo Fisher Scientific), 100 U of RevertAid reverse transcriptase (Thermo Fisher Scientific). The mixture was incubated for 60 min at 42 °C, and the enzyme was inactivated for 10 min at 70 °C

Preparation of the normalization and expression samples. To prepare each sample, two rounds of PCR were performed. For the first round of amplification, we used 600 ng of genomic DNA template (for the normalization sample) or 3 μl of cDNA (for the expression sample), 0.5 μl of 10 μM primers Libr-cDNA-for and Libr-cDNA-A16-rev / Libr-cDNA-A23- rev (see Table 1) for the normalization / expression sample, respectively, 5 μl of 5× Phusion HF buffer (Thermo Fisher Scientific), 2 μl of 2.5 mM dNTPs, and 1.25 U of Phusion HS II DNA polymerase (Thermo Fisher Scientific) in a total volume of 25 μl. The thermal cycle conditions of the first round of PCR were as follows: 98 °C for 1 min, 15 cycles of 98 °C for 30 sec, 70 °C for 30 sec, 72 °C for 30 sec, and a final incubation for 5 min at 72 °C. The second round of amplification was carried out in a volume of 25 μl with the following components: 0.5 μl of the PCR products of the first round, 0.25 μl of 10 μM primers Libr-P5-for and Libr-P7-rev (see Table 1), 5 μl of 5× Phusion HF buffer (Thermo Fisher Scientific), 2 μl of 2.5 mM dNTPs, and 1.25 U of Phusion HotStart II DNA polymerase (Thermo Fisher Scientific). The thermal cycle conditions of the second round of PCR were as follows: 98 °C for 1 min, 23 cycles of 98 °C for 30 sec, 61 °C for 30 sec, 72 °C for 30 sec, and a final incubation for 5 min at 72 °C

Preparation of the mapping sample. Two μg of genomic DNA was incubated with 10 U of DpnII restriction endonuclease (New England Biolabs) at 37 °C for 16 h, then purified using the GeneJET PCR Purification Kit (Thermo Fisher Scientific). 600 ng of the digested genomic DNA was mixed with 4 μl of 100 mM ATP, 2.5 U of T4 DNA ligase (Evrogen) in a total volume of 400 μl. The ligase mixture was incubated for 2 h at room temperature and then for 16 h at 4 °C, followed by enzyme inactivation at 65 °C for 10 min. 100 μl of double-distilled water and 500 μl of a phenol:chloroform solution (1:1 ratio) were added to the ligation reaction, mixed, centrifuged at room temperature for 5 min at 10,000×g, and the upper phase was transferred to a new tube. One-tenth volume of 3M NaOAc (pH 5.5) and 2.5 volumes of 96 % ethyl alcohol were added to the resulting solution, the mixture was incubated for 2 h at –70 °C, then centrifuged for 30 min at 4 °C, 14,000 rpm. The supernatant was removed, the pellet was washed with 750 μl of chilled 70 % ethyl alcohol, and centrifuged for 10 min at 4 °C, 14,000 rpm. The supernatant was removed, the pellet was dried for 15 min at 37 °C and then dissolved in 30 μl of double-distilled water.

Three rounds of PCR were used to prepare the mapping sample. For the first round of amplification, we used 5 μl of purified ligase mixture, 0.5 μl of 10 μM primers PB-outer- F-2 and cDNA-ampl-R (see Table 1), 5 μl of 5× Phusion HF buffer (Thermo Fisher Scientific), 2 μl of 2.5 mM dNTPs, and 1.25 U of Phusion HS II DNA polymerase (Thermo Fisher Scientific) in a total volume of 25 μl. The subsequent rounds of amplification were carried out similarly using (i) primers PB-cDNA-fwd-A7 and InvPCR-F-Nextera2 (see Table 1) and 1 μl of the PCR products of the first round for the second round of amplification and (ii) primers Libr-P5-for and Libr-P7-rev (see Table 1) and 1 μl of the PCR products of the second round for the third round of amplification. The thermal cycle conditions for the first round of PCR were as follows: 98 °C for 1 min, 12 cycles of 98 °C for 30 sec, 65 °C for 30 sec, 72 °C for 2 min, and a final incubation for 5 min at 72 °C. The thermal cycle conditions for the second round of PCR were as follows: 98 °C for 1 min, 12 cycles of 98 °C for 30 sec, 62 °C for 30 sec, 72 °C for 2 min, and a final incubation for 5 min at 72 °C. The thermal cycle conditions for the third round of PCR were as follows: 98 °C for 1 min, 16 cycles of 98 °C for 30 sec, 61 °C for 30 sec, 72 °C for 2 min, and a final incubation for 5 min at 72 °C. Finally, 5 μg of the mapping sample was treated with 10 U of NotI restriction endonuclease (New England Biolabs) in a total volume of 100 μl at 37 °C for 2 h to remove byproducts.

High-throughput sequencing and data analysis. Sequencing of the samples was performed on the Genolab 2 × 75 bp platform (https://genomed.ru/). Demultiplexing of the obtained fastq files using the sabre tool (https://github.com/ najoshi/sabre) resulted in ~4.5, ~1.6 and ~1 million reads for the mapping, normalization, and expression samples, respectively. The quality analysis of the raw data was carried out using the FastQC tool (https://www.bioinformatics.babraham. ac.uk/projects/fastqc/). Next, using the TASK tool (The TRIP Analysis Software Kit, https://trip.nki.nl/), the sequences of reliably identified barcodes, as well as their normalized expression levels and genomic locations in the Chinese hamster genome assemblies CriGri-PICRH-1.0 (GCA_003668045.2) and Cgr1.0 (GCA_000448345.1) were identified. The genome assembly CriGri-PICRH-1.0 is characterized by the presence of extremely long sequences corresponding to all expected chromosomes, and therefore it was used as the “default” genome of CHO cells, whereas the genome assembly Cgr1.0 was previously used to map chromatin types in CHO-K1 cells (Feichtinger et al., 2016). Then, to determine the most reliable (hereafter referred to as filtered) transgenes, the following additional parameters were applied to the TASK output: norm >= 5, reads_r >= 10, freq1_r > 0.60. Data on chromatin types were taken for the Tp0 time point corresponding to the culturing of CHO-K1 cells for 4 h (https://cho-epigenome. boku.ac.at/JB/). A positional weight matrix for genomic sequences overlapping transgene insertion sites was generated using the pLogo application (https://plogo.uconn.edu/) (O’Shea et al., 2013).

## Results and discussion

**Fig. 1.**
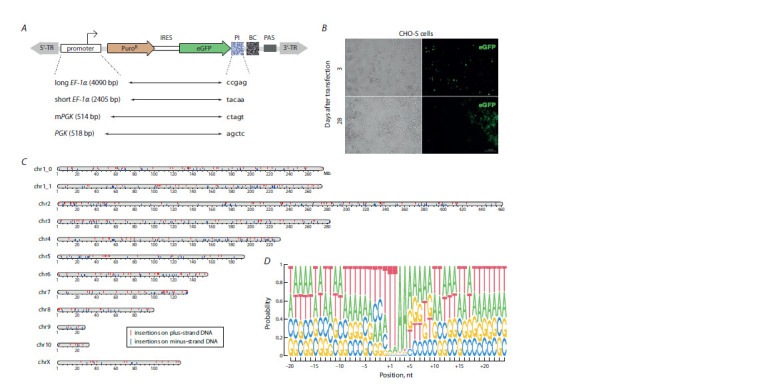
Stable integration of model piggyBac transgenes into the genome of cultured CHO cells. А, Schematic of the barcoded reporter constructs with four different promoters used in the study. 5’-TR and 3’-TR are inverse terminal repeats of the piggyBac transposon; IRES – internal ribosome entry site, PI – promoter index, BC – barcode, PAS – polyadenylation signal. B, CHO cells 3 and 28 days after transfection. C, Distribution of all uniquely mapped transgenes across Chinese hamster chromosomes. Red and blue vertical lines indicate transgene insertions on the plus and minus strand of the genome, respectively. D, Analysis of the genomic motifs, at which integration of all uniquely mapped transgenes occurred. Positions +1…+4 correspond to the sequence that is duplicated upon the piggyBac transposon insertion and flanks the integrated transgene

To study the activity of several promoters in different local chromatin environments in CHO cells in parallel, barcoded model transgenes were constructed based on the piggyBac transposon. The transgenes contained the puromycin-N-acetyltransferase (pac) resistance gene (hereafter PuroR) and the improved green fluorescent protein (eGFP) gene (separated by an IRES element) under the control of the following four promoters: (1) the promoter of the mouse PGK (mPGK) gene, previously used for a similar study on mouse embryonic stem cells (Akhtar et al., 2013), (2) the promoter of the Chinese hamster PGK gene, homologous to the mPGK promoter, (3) full-length (“long”) and (4) truncated (“short”) variants of the Chinese hamster EF-1α gene promoter (Running Deer, Allison, 2004; Orlova et al., 2014; Wang et al., 2017) (Fig. 1, A). Constructs with each individual promoter also contained a specific 5-bp motif (promoter index) immediately before the 18-bp barcode located in the 3′UTR region. The presence of promoter indexes allowed the simultaneous use of all 4 barcoded model transgenes in a single experiment (Gisler et al., 2019) (see Fig. 1, A). Thus, the resulting barcoded plasmid libraries of the constructs with the long EF-1α, short EF-1α, mPGK and PGK promoters were mixed in a molar ratio of 7:7:7:1. The smaller proportion of the latter construct was due to its use in this experiment as a control; we also used the PGK promotercontaining construct to obtain stable transgenic populations of CHO cells upon puromycin selection (the results of that study will be reported elsewhere) and it seemed useful to us to have the technical ability to correctly compare data for such different transgenic populations in the future.

CHO cells (CHO-S subline) were co-transfected with the above-described mixture of model transgenes, as well as a plasmid encoding the piggyBac transposase. Seventy-two h after transfection, the eGFP protein expression was observed in approximately 40 % of the cells (see Fig. 1, B). After that, the cells were cultivated in the absence of any selection for additional 25 days in order to multiply the transgenic cells and to get rid of plasmid DNA molecules that could ultimately contaminate the data. As a result, multiple clones of transgenic cells were observed in the population (see Fig. 1, B).

From the resulting polyclonal population of cells, genomic DNA and total RNA were isolated, on the basis of which the genomic localizations and normalized expression levels of barcoded transgenes were determined. A total of 641 uniquely barcoded and genome-mapped transgenes were identified in the transgenic population. These transgenes were present in more or less expected numbers on all chromosomes of CHO cells (see Fig. 1, C). Analysis of genomic sequences overlapping transgene insertion sites revealed their at-richness, as well as the presence of a central ttaa motif (see Fig. 1, D) specific for the piggyBac transposon (Frase et al., 1996; Li et al., 2013; Chen Q. et al., 2020).

**Fig. 2.**
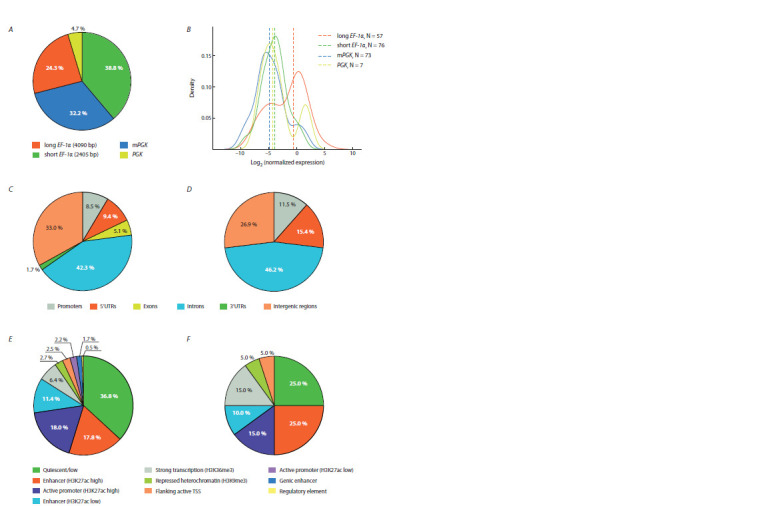
Features of studied transgenes А, Distribution of all identified transgenes across the studied promoters. B, Comparison of promoter activities for the filtered 144 expressed transgenes (see Materials and methods). Dashed vertical lines indicate median normalized expression values. C, D, Distribution of all identified transgenes (C ) and 10 % of the most active filtered transgenes (D) in gene elements (promoters, 5’UTRs, exons, introns, 3’UTRs) as well as intergenic regions. E, F, Distribution of all transgenes (E ) and 10 % of the most active filtered transgenes (F ) in chromatin types of CHO-K1 cells described previously (Feichtinger et al., 2016).

Among the identified transgenes, 38.8 % were with the tacaa promoter index (corresponding to the short EF-1α promoter), 24.3 %, with the ccgag promoter index (corresponding to the long EF-1α promoter), 32.2 %, with the ctagt promoter index (corresponding to the mPGK promoter), and 4.7 %, with the promoter index agctc (corresponding to the Chinese hamster PGK promoter) (Fig. 2, A).

Analysis of the activity of reporter constructs under the control of four different promoters revealed the presence of a large number of silent (i. e., transcriptionally inactive) transgenes with each promoter (Table 2), which is most likely due to the lack of antibiotic selection during generation of the population of transgenic cells.

**Table 2.**
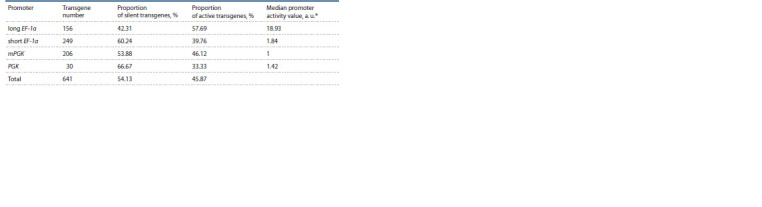
Comparative activity of the studied promoters * a. u. – arbitrary units.

Comparison of promoter activities among the filtered expressed transgenes (144 cases) showed that the main part of highly active reporter constructs is under the control of the full-length variant of the EF-1α gene promoter (see Fig. 2, B, Table 2). Particularly, among 10 % of the most active filtered transgenes, the promoters were distributed as follows: long EF-1α – 70 %, mPGK – 20 %, short EF-1α – 10 %, PGK – 0 %. It is worth noting that due to the small number of transgenes with the PGK promoter, the results on its activity are rather preliminary.

Two thirds of all transgenes were inserted into the genome of CHO cells within genes (which were defined as –1000 bp from the distal transcription initiation site to the transcription termination site), predominantly in introns (42.3 %), promoters (8.5 %) and 5′UTRs (9.4 %) (see Fig. 2, C). It should be noted that promoters were defined as –1000…+100 bp from the transcription start site. Similar patterns of transgene integration based on the piggyBac transposon have been described previously for cell cultures of other species (Ding et al., 2005; Wilson et al., 2007; Galvan et al., 2009; Li et al., 2013). Analysis of 10 % of the most active filtered reporter constructs (21 cases) revealed a ~1.6-fold, ~1.4-fold and ~1.1-fold increase in the proportion of transgenes in 5′UTRs, promoters and introns, respectively (see Fig. 2, D). In addition, it is interesting to note that transgenes were more often located closer to the gene starts than to the gene ends. The median distances from the transgene localization position in the genome to the nearest transcription initiation and termination sites were 11.4 and 20.2 kb, respectively, for the complete set of reporter genes (641 cases). At the same time, for 10 % of the most active filtered transgenes (21 cases), these values were equal to 6.6 and 17.8 kb, respectively.

To study the influence of the local chromatin environment on the activity of reporter genes, previously published data on the distribution of 11 chromatin types in the genome of CHO-K1 cells were used (Feichtinger et al., 2016). Because these data were originally associated with a version of the Chinese hamster genome that is different from that used for the analysis described above (see Materials and methods for details), it was possible to extract chromatin types overlapping positions of transgenes in the genome only for 595 out of 641 transgenes. Among these 595 transgenes, only 39.5 % were localized in inactive chromatin types “Quiescent/low”, “Repressed heterochromatin (H3K9me3)” and “Polycomb repressed regions (H3K27me3)”, which together cover more than 88 % of the genome of Chinese hamster cells (Feichtinger et al., 2016). The remaining 60.5 % of transgenes were localized in various active chromatin types (see Fig. 2, E ).

The most active transgenes were more often localized in regions of the genome associated with the active chromatin types “Enhancer (H3K27ac high)”, “Strong transcription (H3K36me3)” and “Flanking active TSS” as well as with the inactive chromatin type “Repressed heterochromatin (H3K9me3)” (see Fig. 2, F ). The latter rather unexpected observation may be due to the fact that the chromatin types were determined for a different subline of CHO cells.

**Fig. 3.**
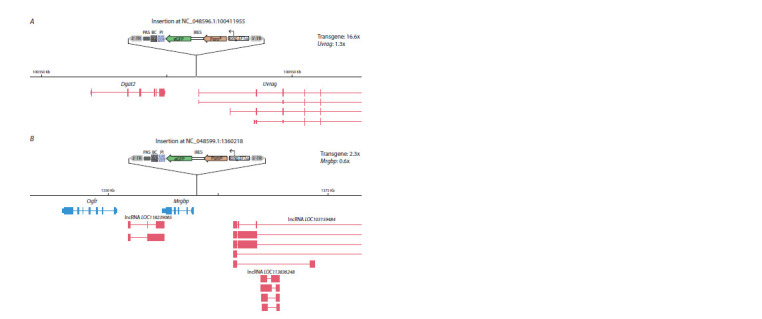
Examples of genomic localization of transgenes with high (A) and medium (B) transcriptional activity. The transgenes are not shown in scale. The activities of the transgenes and the genes nearest to them are indicated relative to the average expression levels of all reporter genes (641 cases) and all endogenous genes, respectively.

Since, as noted above, two thirds of all transgenes were localized within genes (see Fig. 2, C ), it is worth noting that the insertion of a transgene even into an important gene most likely has only a minor effect on cell viability. This is supported by the following two considerations. First, not every insertion of a foreign sequence within a gene significantly disrupts its function. Second, typically, there is another native copy(ies) of the gene in the genome of cultured cells. Together, this ensures the successful survival of transgenic cells in a polyclonal population among non-modified (wild-type) cells. The chances of damaging both alleles of a gene in the experimental setup used are negligible: to achieve that, two transgenes must be integrated into both alleles of the same gene in the same cell. Accordingly, the genomic positions of active transgenes identified in this study may qualify for consideration as promising sites for targeted integration of biotechnological transgenes, even if they are located inside active genes (Fig. 3).

## Conclusion

In a polyclonal population of transgenic CHO cells cultured in the absence of selection pressure, more than half of the model reporter constructs stably integrated into the genome were transcriptionally inactive. Compared to the complete set of transgenes, the most active transgenes were about 1.6 and 1.4 times more frequently localized in promoters and 5′UTRs of genes, respectively. Also, compared to the complete set of transgenes, the most active transgenes were about 2.3 and 1.4 times more frequently localized in the transcriptionally active chromatin types “Strong transcription (H3K36me3)” and “Enhancer (H3K27ac high)”, respectively. Transgenes containing the full-length promoter of the Chinese hamster EF-1α gene were on average the most active ones. At the same time, the median activity of the short variant of the EF- 1α gene promoter was about 10 times lower than the median activity of the full-length promoter of this gene. This can be explained by the presence of important binding sites for transcription factors in the full-length version of the EF-1α gene promoter. Genomic sites of the most active insertions of model transgenes may be of interest for further experiments as promising positions for targeted integration of biotechnological constructs.

## Conflict of interest

The authors declare no conflict of interest.
